# 3′,6′-Bis(diethyl­amino)-2-(2-hydroxy­ethyl­amino)spiro­[isoindoline-1,9′-xanthen]-3-one

**DOI:** 10.1107/S1600536808017108

**Published:** 2008-06-13

**Authors:** Mao-Zhong Tian, Xiao-Jun Peng

**Affiliations:** a College of Chemical Engineering, Shanxi Datong University, Datong 037009, People’s Republic of China; b State Key Laboratory of Fine Chemicals, Dalian University of Technology, 158 Zhongshan Rd, Dalian 116012, People’s Republic of China

## Abstract

In the title mol­ecule, C_30_H_36_N_4_O_3_, the dihedral angle between the planes of the xanthene and spiro­lactam rings systems is 88.69 (4)°. Both C atoms of one of the ethyl groups are disordered over two sites with occupancies 0.72 (2)/0.28 (2). The conformation of the mol­ecule may be influenced by two intra­molecular hydrogen bonds.

## Related literature

For related literature, see: Zhang *et al.* (2007 ▶); Wu *et al.* (2007 ▶); Bae & Tae 2007 ▶).
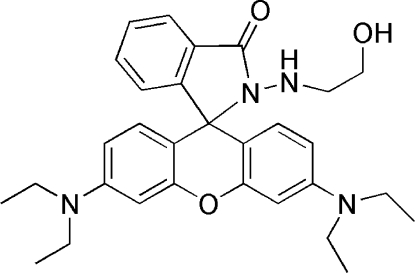


## Experimental

### 

#### Crystal data

C_30_H_36_N_4_O_3_
*M* *_r_* = 500.63
Monoclinic, 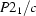

*a* = 12.269 (4) Å
*b* = 12.203 (4) Å
*c* = 18.458 (6) Å
β = 108.127 (5)°
*V* = 2626.4 (15) Å^3^
*Z* = 4
Mo *K*α radiation
μ = 0.08 mm^−1^
*T* = 298 (2) K
0.58 × 0.25 × 0.25 mm

#### Data collection

Bruker SMART APEXII diffractometer
Absorption correction: multi-scan (*SADABS*; Bruker, 2005 ▶) *T*_min_ = 0.849, *T*_max_ = 0.900 (expected range = 0.924–0.979)
17363 measured reflections
4620 independent reflections
2841 reflections with *I* > 2σ(*I*)
*R*_int_ = 0.064

#### Refinement

*R*[*F* ^2^ > 2σ(*F* ^2^)] = 0.050
*wR*(*F* ^2^) = 0.129
*S* = 1.00
4620 reflections
359 parameters
13 restraints
H atoms treated by a mixture of independent and constrained refinement
Δρ_max_ = 0.21 e Å^−3^
Δρ_min_ = −0.20 e Å^−3^

### 

Data collection: *APEX2* (Bruker, 2005 ▶); cell refinement: *SAINT* (Bruker, 2005 ▶); data reduction: *SAINT*; program(s) used to solve structure: *SHELXS97* (Sheldrick, 2008 ▶); program(s) used to refine structure: *SHELXL97* (Sheldrick, 2008 ▶); molecular graphics: *PLATON* (Spek, 2003 ▶); software used to prepare material for publication: *SHELXTL* (Sheldrick, 2008 ▶).

## Supplementary Material

Crystal structure: contains datablocks global, I. DOI: 10.1107/S1600536808017108/lh2636sup1.cif

Structure factors: contains datablocks I. DOI: 10.1107/S1600536808017108/lh2636Isup2.hkl

Additional supplementary materials:  crystallographic information; 3D view; checkCIF report

## Figures and Tables

**Table 1:** Hydrogen-bond geometry (Å, °)

*D*—H⋯*A*	*D*—H	H⋯*A*	*D*⋯*A*	*D*—H⋯*A*
O3—H3*O*⋯O2	1.00 (4)	1.79 (4)	2.780 (3)	172 (4)
N4—H1*N*⋯O2	0.96 (3)	2.45 (2)	2.828 (3)	103 (2)

**Fig. 1.**
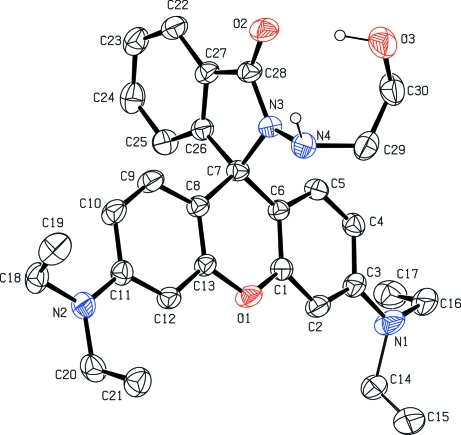
The molecular structure of (I), showing the atom-numbering scheme and displacement ellipsoids at the 30% probability level. H atoms bonded to C atoms have been omitted and the disorder is not shown.

## supplementary crystallographic information

### Comment

Rhodamine dyes are molecules used extensively as fluorescent labeling reagents and dye laser sources because of their excellent photophysical properties, such as long absorption and emission wavelengths elongated to visible region, high fluorescence quantum yield, and large absorption coefficient. (Zhang *et al.*, 2007; Wu *et al.*, 2007; Bae & Tae, 2007). Detailed information on their molecular and crystal structures is necessary to understand their photophysical and photochemical properties. As part of our own work on rhodamine derivatives, we report here the synthesis and crystal structure of the title compound (I).

As shown in Fig.1, the xanthene ring is close to planar with an r.m.s. deviation of 0.089 (9) Å. The lactam moiety of the molecule is oriented nearly orthogonal to the xanthene moiety *i.e.* the dihedral angle between the planes of the xanthene and the spirolactam rings systems is 88.69 (4)°.

### Experimental

Sodium borohydride (15.2 mg, 0.4 mmol) was slowly added to a solution of compound 3',6'-Bis(diethylamino)-2-(2-oxoethylideneamino)spiro [isoindoline-1,9'-xanthen]-3-one (150 mg, 0.3 mmol) in ethanol (20 ml). The reaction mixture was stirred for 2 h at room temperature and solvent was totally removed under reduced pressure. The crude product was dissolved in CH_2_Cl_2_ (20 ml) and 3 ml of an aqueous solution of K_2_CO_3_ was added. The organic layer was dried over MgSO_4_. After filtration, the solvent was removed under reduced pressure. The residue was placed on a silica gel column (200–300 mesh). The column was eluted with a mixture (2:1, *v/v*) of petroleum spirit/ethyl acetate, to give 136 mg of the title compound (90%). Crystals were grown by dissolving the compound in CH_2_Cl_2_ and slowly diffusing n-hexane into the solution.

### Refinement

H atoms bonded to Catoms were positioned geometrically (C—H = 0.93–0.97 Å) and refined as riding, with *U*_iso_(H) = 1.2*U*_eq_(C) or 1.5*U*_eq_(methyl C). The positional parameters of the H atoms bonded to N and O were refined independently with *U*_iso_(H) = 1.5*U*_eq_(N,*O*).

### Crystal data

**Table:**

C_30_H_36_N_4_O_3_	*F*_000_ = 1072
*M**_r_* = 500.63	*D*_x_ = 1.266 Mg m^−^^3^
Monoclinic, *P*2_1_/*c*	Mo *K*α radiation λ = 0.71073 Å
Hall symbol: -P 2ybc	Cell parameters from 3630 reflections
*a* = 12.269 (4) Å	θ = 2.0–25.0º
*b* = 12.203 (4) Å	µ = 0.08 mm^−^^1^
*c* = 18.458 (6) Å	*T* = 298 (2) K
β = 108.127 (5)º	Block, white
*V* = 2626.4 (15) Å^3^	0.58 × 0.25 × 0.25 mm
*Z* = 4	

### Data collection

**Table:**

Bruker SMART APEXII diffractometer	4620 independent reflections
Radiation source: fine-focus sealed tube	2841 reflections with *I* > 2σ(*I*)
Monochromator: graphite	*R*_int_ = 0.064
*T* = 298(2) K	θ_max_ = 25.0º
φ and ω scans	θ_min_ = 2.0º
Absorption correction: multi-scan(SADABS; Bruker, 2005)	*h* = −14→14
*T*_min_ = 0.849, *T*_max_ = 0.900	*k* = −14→14
17363 measured reflections	*l* = −21→21

### Refinement

**Table:**

Refinement on *F*^2^	Secondary atom site location: difference Fourier map
Least-squares matrix: full	Hydrogen site location: inferred from neighbouring sites
*R*[*F*^2^ > 2σ(*F*^2^)] = 0.050	H atoms treated by a mixture of independent and constrained refinement
*wR*(*F*^2^) = 0.129	*w* = 1/[σ^2^(*F*_o_^2^) + (0.0755*P*)^2^] where *P* = (*F*_o_^2^ + 2*F*_c_^2^)/3
*S* = 1.00	(Δ/σ)_max_ = 0.002
4620 reflections	Δρ_max_ = 0.21 e Å^−^^3^
359 parameters	Δρ_min_ = −0.20 e Å^−^^3^
13 restraints	Extinction correction: SHELXL97 (Sheldrick, 2008), Fc^*^=kFc[1+0.001xFc^2^λ^3^/sin(2θ)]^-1/4^
Primary atom site location: structure-invariant direct methods	Extinction coefficient: 0.026 (4)

### Special details

**Table:**

Experimental. ^1^H NMR (CDCl_3_, 400MHz, Me_4_Si): δ 7.91 (d, 1H, *J*=6.4 Hz, C_6_H_4_), 7.52-7.47 (m, 2H, C_6_H_4_), 7.15 (d, 1H, *J*= 6.4 Hz), 6.41 (m, 4H, Xanthene-H), 6.26 (dd, 2H, *J*= 8.8 Hz, *J*= 2.4 Hz, Xanthene-H), 4.65 (t, 1H, *J*= 7.2 Hz, NH), 4.45 (t, 1H, *J*= 6.0 Hz, OH), 3.36-3.31 (m, 10H, CH_2_O, CH_2_), 2.46-2.45 (m, 2H, CH_2_N), 1.16 (t, 12H, *J*=6.8 Hz, CH_3_); ^13^C NMR (CDCl_3_, 100MHz, Me_4_Si): δ 168.31, 164.31, 153.97, 151.59, 149.05, 133.17, 129.99, 128.51, 123.08, 124.20, 107.97, 105.29, 97.98, 66.39, 58.76, 52.83, 44.51, 12.75. HRMS(ESI): calcd for C_30_H_36_N_4_O_3_ [M+Na]^+^ 523.2685; found 523.2671.
Geometry. All e.s.d.'s (except the e.s.d. in the dihedral angle between two l.s. planes) are estimated using the full covariance matrix. The cell e.s.d.'s are taken into account individually in the estimation of e.s.d.'s in distances, angles and torsion angles; correlations between e.s.d.'s in cell parameters are only used when they are defined by crystal symmetry. An approximate (isotropic) treatment of cell e.s.d.'s is used for estimating e.s.d.'s involving l.s. planes.
Refinement. Refinement of *F*^2^ against ALL reflections. The weighted *R*-factor *wR* and goodness of fit *S* are based on *F*^2^, conventional *R*-factors *R* are based on *F*, with *F* set to zero for negative *F*^2^. The threshold expression of *F*^2^ > 2σ(*F*^2^) is used only for calculating *R*-factors(gt) *etc*. and is not relevant to the choice of reflections for refinement. *R*-factors based on *F*^2^ are statistically about twice as large as those based on *F*, and *R*- factors based on ALL data will be even larger.

### Fractional atomic coordinates and isotropic or equivalent isotropic displacement parameters (Å^2^)

**Table:**

	*x*	*y*	*z*	*U*_iso_*/*U*_eq_	Occ. (<1)
O1	0.02779 (11)	0.63551 (12)	0.15665 (9)	0.0590 (5)	
O2	−0.40178 (12)	0.91274 (15)	0.13067 (9)	0.0670 (5)	
O3	−0.34132 (15)	0.9162 (2)	0.28884 (13)	0.0941 (7)	
H3O	−0.370 (3)	0.915 (3)	0.232 (2)	0.141*	
N1	0.31949 (16)	0.86643 (19)	0.30367 (13)	0.0770 (7)	
N2	−0.19898 (16)	0.38340 (17)	−0.02009 (11)	0.0620 (5)	
N3	−0.22666 (13)	0.83188 (15)	0.15192 (9)	0.0443 (4)	
N4	−0.23247 (17)	0.76085 (17)	0.20976 (11)	0.0570 (5)	
H1N	−0.313 (2)	0.749 (2)	0.2013 (14)	0.085*	
C1	0.05554 (16)	0.74041 (19)	0.18219 (11)	0.0459 (5)	
C2	0.16682 (17)	0.7528 (2)	0.22812 (12)	0.0551 (6)	
H2A	0.2155	0.6925	0.2386	0.066*	
C3	0.20776 (17)	0.8541 (2)	0.25922 (13)	0.0525 (6)	
C4	0.13007 (17)	0.94070 (19)	0.24316 (12)	0.0488 (6)	
H4A	0.1530	1.0093	0.2643	0.059*	
C5	0.02040 (17)	0.92561 (18)	0.19652 (12)	0.0468 (5)	
H5A	−0.0292	0.9853	0.1863	0.056*	
C6	−0.02056 (15)	0.82580 (18)	0.16369 (11)	0.0407 (5)	
C7	−0.14135 (15)	0.81057 (17)	0.11196 (11)	0.0417 (5)	
C8	−0.15672 (16)	0.69727 (18)	0.07841 (11)	0.0439 (5)	
C9	−0.25529 (17)	0.6667 (2)	0.02086 (13)	0.0570 (6)	
H9A	−0.3130	0.7186	0.0030	0.068*	
C10	−0.27173 (19)	0.5653 (2)	−0.01066 (13)	0.0593 (6)	
H10A	−0.3398	0.5491	−0.0487	0.071*	
C11	−0.18663 (17)	0.48494 (19)	0.01373 (12)	0.0501 (6)	
C12	−0.08854 (17)	0.51411 (19)	0.07132 (12)	0.0494 (6)	
H12A	−0.0307	0.4626	0.0898	0.059*	
C13	−0.07482 (16)	0.61794 (18)	0.10178 (11)	0.0436 (5)	
C14	0.4083 (4)	0.7842 (6)	0.2970 (4)	0.062 (2)	0.720 (17)
H14A	0.3815	0.7481	0.2478	0.074*	0.720 (17)
H14B	0.4792	0.8221	0.3002	0.074*	0.720 (17)
C15	0.4301 (7)	0.7010 (7)	0.3586 (4)	0.099 (2)	0.720 (17)
H15A	0.4863	0.6496	0.3533	0.149*	0.720 (17)
H15B	0.3601	0.6631	0.3550	0.149*	0.720 (17)
H15C	0.4580	0.7367	0.4072	0.149*	0.720 (17)
C14A	0.3796 (13)	0.7676 (16)	0.3463 (9)	0.074 (7)	0.280 (17)
H14C	0.4289	0.7869	0.3969	0.088*	0.280 (17)
H14D	0.3254	0.7120	0.3505	0.088*	0.280 (17)
C15A	0.4469 (17)	0.731 (2)	0.2969 (13)	0.111 (7)	0.280 (17)
H15D	0.4904	0.6668	0.3187	0.166*	0.280 (17)
H15E	0.4983	0.7881	0.2928	0.166*	0.280 (17)
H15F	0.3959	0.7135	0.2472	0.166*	0.280 (17)
C16	0.3656 (2)	0.9732 (2)	0.33183 (16)	0.0739 (8)	
H16A	0.4332	0.9633	0.3757	0.089*	
H16B	0.3092	1.0128	0.3485	0.089*	
C17	0.3969 (2)	1.0400 (3)	0.27363 (18)	0.0852 (9)	
H17A	0.4269	1.1094	0.2953	0.128*	
H17B	0.3299	1.0517	0.2305	0.128*	
H17C	0.4539	1.0020	0.2576	0.128*	
C18	−0.3044 (2)	0.3522 (2)	−0.07672 (14)	0.0690 (7)	
H18A	−0.2890	0.2907	−0.1054	0.083*	
H18B	−0.3301	0.4127	−0.1120	0.083*	
C19	−0.3997 (2)	0.3211 (3)	−0.04619 (17)	0.0877 (9)	
H19A	−0.4665	0.3023	−0.0877	0.132*	
H19B	−0.4170	0.3817	−0.0185	0.132*	
H19C	−0.3766	0.2592	−0.0127	0.132*	
C20	−0.1110 (2)	0.2997 (2)	0.00744 (14)	0.0665 (7)	
H20A	−0.0363	0.3339	0.0183	0.080*	
H20B	−0.1183	0.2465	−0.0328	0.080*	
C21	−0.1165 (2)	0.2407 (2)	0.07736 (15)	0.0744 (8)	
H21A	−0.0561	0.1874	0.0921	0.112*	
H21B	−0.1893	0.2047	0.0668	0.112*	
H21C	−0.1075	0.2924	0.1180	0.112*	
C22	−0.34216 (19)	1.0120 (2)	−0.00789 (14)	0.0593 (6)	
H22A	−0.4118	1.0420	−0.0078	0.071*	
C23	−0.2949 (2)	1.0373 (2)	−0.06426 (14)	0.0654 (7)	
H23A	−0.3340	1.0832	−0.1041	0.078*	
C24	−0.1899 (2)	0.9951 (2)	−0.06186 (14)	0.0692 (7)	
H24A	−0.1574	1.0154	−0.0991	0.083*	
C25	−0.13217 (19)	0.9237 (2)	−0.00578 (13)	0.0571 (6)	
H25A	−0.0617	0.8949	−0.0051	0.068*	
C26	−0.18049 (16)	0.89571 (18)	0.04935 (12)	0.0455 (5)	
C27	−0.28307 (16)	0.94066 (18)	0.04841 (12)	0.0482 (6)	
C28	−0.31390 (17)	0.89661 (19)	0.11342 (12)	0.0499 (6)	
C29	−0.1807 (2)	0.8051 (3)	0.28589 (13)	0.0694 (8)	
H29A	−0.0986	0.8101	0.2953	0.083*	
H29B	−0.1933	0.7534	0.3224	0.083*	
C30	−0.2233 (2)	0.9153 (3)	0.30112 (16)	0.0804 (9)	
H30A	−0.1842	0.9361	0.3535	0.097*	
H30B	−0.2048	0.9693	0.2682	0.097*	

### Atomic displacement parameters (Å^2^)

**Table:**

	*U*^11^	*U*^22^	*U*^33^	*U*^12^	*U*^13^	*U*^23^
O1	0.0457 (8)	0.0423 (10)	0.0714 (10)	0.0041 (7)	−0.0072 (7)	−0.0086 (8)
O2	0.0416 (8)	0.0841 (14)	0.0751 (11)	0.0098 (8)	0.0178 (8)	0.0037 (9)
O3	0.0625 (11)	0.143 (2)	0.0822 (13)	0.0030 (11)	0.0306 (10)	−0.0282 (13)
N1	0.0511 (11)	0.0595 (16)	0.0937 (16)	0.0007 (10)	−0.0163 (11)	−0.0167 (13)
N2	0.0701 (12)	0.0476 (13)	0.0588 (13)	−0.0039 (10)	0.0065 (10)	−0.0138 (10)
N3	0.0412 (9)	0.0439 (11)	0.0479 (10)	0.0031 (8)	0.0141 (7)	0.0043 (9)
N4	0.0620 (11)	0.0576 (14)	0.0535 (12)	0.0019 (10)	0.0211 (10)	0.0056 (10)
C1	0.0424 (11)	0.0430 (15)	0.0490 (13)	−0.0023 (10)	0.0093 (9)	−0.0057 (10)
C2	0.0467 (12)	0.0491 (16)	0.0587 (14)	0.0057 (11)	0.0009 (10)	−0.0040 (12)
C3	0.0426 (11)	0.0570 (17)	0.0505 (13)	−0.0034 (11)	0.0039 (9)	−0.0047 (11)
C4	0.0479 (12)	0.0478 (15)	0.0502 (13)	−0.0037 (10)	0.0146 (10)	−0.0092 (11)
C5	0.0431 (11)	0.0436 (15)	0.0547 (13)	0.0025 (10)	0.0167 (10)	−0.0038 (11)
C6	0.0376 (10)	0.0411 (14)	0.0427 (11)	0.0011 (9)	0.0115 (8)	0.0000 (10)
C7	0.0368 (10)	0.0389 (14)	0.0484 (12)	−0.0003 (9)	0.0117 (9)	−0.0015 (10)
C8	0.0395 (10)	0.0429 (14)	0.0467 (12)	0.0005 (10)	0.0097 (9)	−0.0005 (10)
C9	0.0465 (12)	0.0463 (16)	0.0650 (15)	0.0033 (11)	−0.0019 (10)	−0.0006 (12)
C10	0.0540 (13)	0.0500 (16)	0.0581 (15)	−0.0037 (12)	−0.0055 (11)	−0.0046 (12)
C11	0.0545 (13)	0.0475 (15)	0.0454 (13)	−0.0040 (11)	0.0115 (10)	−0.0019 (11)
C12	0.0487 (12)	0.0407 (15)	0.0538 (14)	0.0038 (10)	0.0086 (10)	−0.0014 (11)
C13	0.0382 (10)	0.0451 (14)	0.0430 (12)	−0.0011 (10)	0.0061 (9)	−0.0005 (10)
C14	0.039 (2)	0.073 (4)	0.067 (3)	−0.001 (2)	0.007 (2)	−0.008 (3)
C15	0.116 (4)	0.091 (5)	0.083 (4)	0.042 (4)	0.021 (3)	0.013 (3)
C14A	0.058 (8)	0.085 (14)	0.068 (9)	−0.009 (8)	0.006 (6)	−0.020 (7)
C15A	0.089 (11)	0.114 (16)	0.137 (16)	0.022 (9)	0.046 (10)	−0.030 (12)
C16	0.0539 (13)	0.074 (2)	0.0739 (18)	−0.0028 (13)	−0.0099 (13)	−0.0210 (16)
C17	0.0611 (16)	0.084 (2)	0.109 (2)	−0.0023 (15)	0.0243 (16)	−0.0150 (19)
C18	0.0865 (17)	0.0548 (18)	0.0527 (15)	−0.0111 (14)	0.0029 (13)	−0.0077 (12)
C19	0.0829 (18)	0.075 (2)	0.092 (2)	−0.0107 (16)	0.0089 (16)	0.0116 (17)
C20	0.0777 (16)	0.0508 (17)	0.0700 (17)	−0.0048 (13)	0.0216 (13)	−0.0173 (13)
C21	0.0750 (17)	0.071 (2)	0.0689 (17)	−0.0050 (14)	0.0101 (13)	−0.0033 (15)
C22	0.0542 (13)	0.0487 (16)	0.0643 (16)	0.0053 (11)	0.0026 (12)	0.0008 (13)
C23	0.0765 (17)	0.0556 (17)	0.0541 (15)	−0.0006 (13)	0.0058 (13)	0.0085 (13)
C24	0.0820 (17)	0.071 (2)	0.0526 (15)	−0.0097 (15)	0.0179 (13)	0.0046 (14)
C25	0.0563 (13)	0.0583 (17)	0.0585 (15)	0.0003 (12)	0.0206 (11)	0.0047 (13)
C26	0.0426 (11)	0.0405 (14)	0.0485 (13)	−0.0011 (9)	0.0069 (9)	−0.0037 (10)
C27	0.0421 (11)	0.0428 (14)	0.0529 (14)	−0.0006 (10)	0.0048 (9)	0.0013 (11)
C28	0.0363 (11)	0.0513 (15)	0.0580 (14)	0.0023 (10)	0.0086 (10)	−0.0027 (11)
C29	0.0540 (13)	0.103 (2)	0.0488 (15)	0.0018 (14)	0.0126 (11)	0.0089 (15)
C30	0.0612 (15)	0.114 (3)	0.0668 (17)	−0.0129 (16)	0.0209 (13)	−0.0303 (17)

### Geometric parameters (Å, °)

**Table:**

O1—C13	1.365 (2)	C15—H15B	0.9600
O1—C1	1.370 (3)	C15—H15C	0.9600
O2—C28	1.231 (2)	C14A—C15A	1.48 (4)
O3—C30	1.394 (3)	C14A—H14C	0.9700
O3—H3O	0.99 (4)	C14A—H14D	0.9700
N1—C3	1.369 (3)	C15A—H15D	0.9600
N1—C16	1.450 (3)	C15A—H15E	0.9600
N1—C14A	1.50 (2)	C15A—H15F	0.9600
N1—C14	1.514 (8)	C16—C17	1.491 (4)
N2—C11	1.374 (3)	C16—H16A	0.9700
N2—C18	1.438 (3)	C16—H16B	0.9700
N2—C20	1.457 (3)	C17—H17A	0.9600
N3—C28	1.342 (3)	C17—H17B	0.9600
N3—N4	1.394 (2)	C17—H17C	0.9600
N3—C7	1.479 (2)	C18—C19	1.496 (4)
N4—C29	1.454 (3)	C18—H18A	0.9700
N4—H1N	0.96 (3)	C18—H18B	0.9700
C1—C6	1.370 (3)	C19—H19A	0.9600
C1—C2	1.373 (3)	C19—H19B	0.9600
C2—C3	1.389 (3)	C19—H19C	0.9600
C2—H2A	0.9300	C20—C21	1.497 (4)
C3—C4	1.392 (3)	C20—H20A	0.9700
C4—C5	1.365 (3)	C20—H20B	0.9700
C4—H4A	0.9300	C21—H21A	0.9600
C5—C6	1.383 (3)	C21—H21B	0.9600
C5—H5A	0.9300	C21—H21C	0.9600
C6—C7	1.504 (3)	C22—C23	1.375 (3)
C7—C8	1.503 (3)	C22—C27	1.376 (3)
C7—C26	1.517 (3)	C22—H22A	0.9300
C8—C13	1.365 (3)	C23—C24	1.375 (3)
C8—C9	1.389 (3)	C23—H23A	0.9300
C9—C10	1.355 (3)	C24—C25	1.370 (3)
C9—H9A	0.9300	C24—H24A	0.9300
C10—C11	1.401 (3)	C25—C26	1.371 (3)
C10—H10A	0.9300	C25—H25A	0.9300
C11—C12	1.382 (3)	C26—C27	1.368 (3)
C12—C13	1.375 (3)	C27—C28	1.468 (3)
C12—H12A	0.9300	C29—C30	1.501 (4)
C14—C15	1.485 (13)	C29—H29A	0.9700
C14—H14A	0.9700	C29—H29B	0.9700
C14—H14B	0.9700	C30—H30A	0.9700
C15—H15A	0.9600	C30—H30B	0.9700
			
C13—O1—C1	118.37 (16)	H14C—C14A—H14D	109.3
C30—O3—H3O	101 (2)	C14A—C15A—H15D	109.5
C3—N1—C16	121.3 (2)	C14A—C15A—H15E	109.5
C3—N1—C14A	117.6 (5)	H15D—C15A—H15E	109.5
C16—N1—C14A	117.4 (4)	C14A—C15A—H15F	109.5
C3—N1—C14	119.3 (2)	H15D—C15A—H15F	109.5
C16—N1—C14	114.5 (2)	H15E—C15A—H15F	109.5
C14A—N1—C14	42.5 (6)	N1—C16—C17	113.1 (2)
C11—N2—C18	121.1 (2)	N1—C16—H16A	109.0
C11—N2—C20	120.56 (19)	C17—C16—H16A	109.0
C18—N2—C20	117.9 (2)	N1—C16—H16B	109.0
C28—N3—N4	123.30 (17)	C17—C16—H16B	109.0
C28—N3—C7	114.26 (17)	H16A—C16—H16B	107.8
N4—N3—C7	119.03 (16)	C16—C17—H17A	109.5
N3—N4—C29	113.5 (2)	C16—C17—H17B	109.5
N3—N4—H1N	105.4 (16)	H17A—C17—H17B	109.5
C29—N4—H1N	109.4 (15)	C16—C17—H17C	109.5
C6—C1—O1	123.23 (17)	H17A—C17—H17C	109.5
C6—C1—C2	122.7 (2)	H17B—C17—H17C	109.5
O1—C1—C2	114.11 (19)	N2—C18—C19	115.1 (2)
C1—C2—C3	121.0 (2)	N2—C18—H18A	108.5
C1—C2—H2A	119.5	C19—C18—H18A	108.5
C3—C2—H2A	119.5	N2—C18—H18B	108.5
N1—C3—C2	120.7 (2)	C19—C18—H18B	108.5
N1—C3—C4	122.4 (2)	H18A—C18—H18B	107.5
C2—C3—C4	116.86 (19)	C18—C19—H19A	109.5
C5—C4—C3	120.5 (2)	C18—C19—H19B	109.5
C5—C4—H4A	119.8	H19A—C19—H19B	109.5
C3—C4—H4A	119.8	C18—C19—H19C	109.5
C4—C5—C6	123.2 (2)	H19A—C19—H19C	109.5
C4—C5—H5A	118.4	H19B—C19—H19C	109.5
C6—C5—H5A	118.4	N2—C20—C21	114.2 (2)
C1—C6—C5	115.73 (18)	N2—C20—H20A	108.7
C1—C6—C7	121.66 (19)	C21—C20—H20A	108.7
C5—C6—C7	122.61 (18)	N2—C20—H20B	108.7
N3—C7—C8	110.47 (16)	C21—C20—H20B	108.7
N3—C7—C6	111.84 (16)	H20A—C20—H20B	107.6
C8—C7—C6	110.36 (16)	C20—C21—H21A	109.5
N3—C7—C26	98.86 (15)	C20—C21—H21B	109.5
C8—C7—C26	110.30 (17)	H21A—C21—H21B	109.5
C6—C7—C26	114.52 (17)	C20—C21—H21C	109.5
C13—C8—C9	115.6 (2)	H21A—C21—H21C	109.5
C13—C8—C7	122.46 (18)	H21B—C21—H21C	109.5
C9—C8—C7	121.98 (19)	C23—C22—C27	117.8 (2)
C10—C9—C8	123.4 (2)	C23—C22—H22A	121.1
C10—C9—H9A	118.3	C27—C22—H22A	121.1
C8—C9—H9A	118.3	C24—C23—C22	120.2 (2)
C9—C10—C11	120.3 (2)	C24—C23—H23A	119.9
C9—C10—H10A	119.8	C22—C23—H23A	119.9
C11—C10—H10A	119.8	C25—C24—C23	121.5 (2)
N2—C11—C12	121.9 (2)	C25—C24—H24A	119.3
N2—C11—C10	121.3 (2)	C23—C24—H24A	119.3
C12—C11—C10	116.8 (2)	C24—C25—C26	118.6 (2)
C13—C12—C11	121.2 (2)	C24—C25—H25A	120.7
C13—C12—H12A	119.4	C26—C25—H25A	120.7
C11—C12—H12A	119.4	C27—C26—C25	119.9 (2)
O1—C13—C8	122.78 (19)	C27—C26—C7	110.87 (18)
O1—C13—C12	114.55 (18)	C25—C26—C7	128.97 (19)
C8—C13—C12	122.67 (19)	C26—C27—C22	122.0 (2)
C15—C14—N1	110.7 (7)	C26—C27—C28	108.24 (18)
C15—C14—H14A	109.5	C22—C27—C28	129.8 (2)
N1—C14—H14A	109.5	O2—C28—N3	125.1 (2)
C15—C14—H14B	109.5	O2—C28—C27	128.5 (2)
N1—C14—H14B	109.5	N3—C28—C27	106.41 (17)
H14A—C14—H14B	108.1	N4—C29—C30	116.2 (2)
C14—C15—H15A	109.5	N4—C29—H29A	108.2
C14—C15—H15B	109.5	C30—C29—H29A	108.2
H15A—C15—H15B	109.5	N4—C29—H29B	108.2
C14—C15—H15C	109.5	C30—C29—H29B	108.2
H15A—C15—H15C	109.5	H29A—C29—H29B	107.4
H15B—C15—H15C	109.5	O3—C30—C29	112.4 (2)
C15A—C14A—N1	101.4 (18)	O3—C30—H30A	109.1
C15A—C14A—H14C	111.5	C29—C30—H30A	109.1
N1—C14A—H14C	111.5	O3—C30—H30B	109.1
C15A—C14A—H14D	111.5	C29—C30—H30B	109.1
N1—C14A—H14D	111.5	H30A—C30—H30B	107.9
			
C28—N3—N4—C29	−98.3 (2)	N2—C11—C12—C13	176.4 (2)
C7—N3—N4—C29	103.7 (2)	C10—C11—C12—C13	−1.3 (3)
C13—O1—C1—C6	9.6 (3)	C1—O1—C13—C8	−8.6 (3)
C13—O1—C1—C2	−170.31 (18)	C1—O1—C13—C12	170.74 (18)
C6—C1—C2—C3	0.7 (4)	C9—C8—C13—O1	178.64 (18)
O1—C1—C2—C3	−179.3 (2)	C7—C8—C13—O1	−0.8 (3)
C16—N1—C3—C2	175.4 (2)	C9—C8—C13—C12	−0.6 (3)
C14A—N1—C3—C2	−27.0 (8)	C7—C8—C13—C12	179.92 (19)
C14—N1—C3—C2	21.6 (5)	C11—C12—C13—O1	−178.19 (19)
C16—N1—C3—C4	−4.9 (4)	C11—C12—C13—C8	1.1 (3)
C14A—N1—C3—C4	152.7 (8)	C3—N1—C14—C15	−98.4 (4)
C14—N1—C3—C4	−158.7 (4)	C16—N1—C14—C15	106.1 (4)
C1—C2—C3—N1	−178.6 (2)	C14A—N1—C14—C15	1.5 (7)
C1—C2—C3—C4	1.6 (3)	C3—N1—C14A—C15A	100.1 (9)
N1—C3—C4—C5	177.9 (2)	C16—N1—C14A—C15A	−101.4 (9)
C2—C3—C4—C5	−2.4 (3)	C14—N1—C14A—C15A	−4.3 (8)
C3—C4—C5—C6	0.9 (3)	C3—N1—C16—C17	−79.7 (3)
O1—C1—C6—C5	177.82 (18)	C14A—N1—C16—C17	122.7 (8)
C2—C1—C6—C5	−2.2 (3)	C14—N1—C16—C17	75.3 (4)
O1—C1—C6—C7	−1.2 (3)	C11—N2—C18—C19	−77.1 (3)
C2—C1—C6—C7	178.74 (19)	C20—N2—C18—C19	95.2 (3)
C4—C5—C6—C1	1.5 (3)	C11—N2—C20—C21	79.8 (3)
C4—C5—C6—C7	−179.54 (19)	C18—N2—C20—C21	−92.5 (3)
C28—N3—C7—C8	−103.7 (2)	C27—C22—C23—C24	−2.3 (4)
N4—N3—C7—C8	56.2 (2)	C22—C23—C24—C25	2.8 (4)
C28—N3—C7—C6	132.94 (19)	C23—C24—C25—C26	−0.9 (4)
N4—N3—C7—C6	−67.2 (2)	C24—C25—C26—C27	−1.4 (4)
C28—N3—C7—C26	11.9 (2)	C24—C25—C26—C7	172.8 (2)
N4—N3—C7—C26	171.83 (17)	N3—C7—C26—C27	−9.7 (2)
C1—C6—C7—N3	116.1 (2)	C8—C7—C26—C27	106.1 (2)
C5—C6—C7—N3	−62.9 (3)	C6—C7—C26—C27	−128.69 (19)
C1—C6—C7—C8	−7.3 (3)	N3—C7—C26—C25	175.7 (2)
C5—C6—C7—C8	173.74 (18)	C8—C7—C26—C25	−68.6 (3)
C1—C6—C7—C26	−132.5 (2)	C6—C7—C26—C25	56.6 (3)
C5—C6—C7—C26	48.6 (3)	C25—C26—C27—C22	1.8 (3)
N3—C7—C8—C13	−115.9 (2)	C7—C26—C27—C22	−173.42 (19)
C6—C7—C8—C13	8.3 (3)	C25—C26—C27—C28	−179.9 (2)
C26—C7—C8—C13	135.9 (2)	C7—C26—C27—C28	4.9 (2)
N3—C7—C8—C9	64.7 (3)	C23—C22—C27—C26	0.1 (3)
C6—C7—C8—C9	−171.09 (18)	C23—C22—C27—C28	−177.8 (2)
C26—C7—C8—C9	−43.6 (3)	N4—N3—C28—O2	10.5 (3)
C13—C8—C9—C10	0.5 (3)	C7—N3—C28—O2	169.5 (2)
C7—C8—C9—C10	179.9 (2)	N4—N3—C28—C27	−168.71 (18)
C8—C9—C10—C11	−0.8 (4)	C7—N3—C28—C27	−9.8 (2)
C18—N2—C11—C12	176.2 (2)	C26—C27—C28—O2	−176.5 (2)
C20—N2—C11—C12	4.1 (3)	C22—C27—C28—O2	1.7 (4)
C18—N2—C11—C10	−6.1 (3)	C26—C27—C28—N3	2.8 (2)
C20—N2—C11—C10	−178.3 (2)	C22—C27—C28—N3	−179.1 (2)
C9—C10—C11—N2	−176.6 (2)	N3—N4—C29—C30	53.9 (3)
C9—C10—C11—C12	1.2 (4)	N4—C29—C30—O3	57.8 (3)

### Hydrogen-bond geometry (Å, °)

**Table:**

*D*—H···*A*	*D*—H	H···*A*	*D*···*A*	*D*—H···*A*
O3—H3O···O2	1.00 (4)	1.79 (4)	2.780 (3)	172 (4)
N4—H1N···O2	0.96 (3)	2.45 (2)	2.828 (3)	103 (2)
